# Proteomic analyses of limbic regions in neonatal male, female and androgen receptor knockout mice

**DOI:** 10.1186/s12868-016-0332-1

**Published:** 2017-01-05

**Authors:** Anna Zettergren, Sara Karlsson, Erik Studer, Anna Sarvimäki, Petronella Kettunen, Annika Thorsell, Carina Sihlbom, Lars Westberg

**Affiliations:** 1 Department of Pharmacology, Institute of Neuroscience and Physiology, Sahlgrenska Academy, University of Gothenburg, POB 431, 405 30 Göteborg, Sweden; 2 Department of Psychiatry and Neurochemistry, Institute of Neuroscience and Physiology, Sahlgrenska Academy, University of Gothenburg, Göteborg, Sweden; 3 Department of Neuropathology, Nuffield Department of Clinical Neurosciences, University of Oxford, John Radcliffe Hospital, Oxford, UK; 4 The Proteomics Core Facility, Sahlgrenska Academy, University of Gothenburg, Göteborg, Sweden

**Keywords:** Proteomics, Sex differences, Androgen receptor, Neonatal, Amygdala, Hypothalamus

## Abstract

**Background:**

It is well-established that organizational effects of sex steroids during early development are fundamental for sex-typical displays of, for example, mating and aggressive behaviors in rodents and other species. Male and female brains are known to differ with respect to neuronal morphology in particular regions of the brain, including the number and size of neurons, and the density and length of dendrites in nuclei of hypothalamus and amygdala. The aim of the present study was to use global proteomics to identify proteins differentially expressed in hypothalamus/amygdala during early development (postnatal day 8) of male, female and conditional androgen receptor knockout (AR^NesDel^) male mice, lacking androgen receptors specifically in the brain. Furthermore, verification of selected sexually dimorphic proteins was performed using targeted proteomics.

**Results:**

Our proteomic approach, iTRAQ, allowed us to investigate expression differences in the 2998 most abundantly expressed proteins in our dissected tissues. Approximately 170 proteins differed between the sexes, and 38 proteins between AR^NesDel^ and control males (p < 0.05). In line with previous explorative studies of sexually dimorphic gene expression we mainly detected subtle protein expression differences (fold changes <1.3). The protein MARCKS (myristoylated alanine rich C kinase substrate), having the largest fold change of the proteins selected from the iTRAQ analyses and of known importance for synaptic transmission and dendritic branching, was confirmed by targeted proteomics as differentially expressed between the sexes.

**Conclusions:**

Overall, our results provide solid evidence that a large number of proteins show sex differences in their brain expression and could potentially be involved in brain sexual differentiation. Furthermore, our finding of a sexually dimorphic expression of MARCKS in the brain during development warrants further investigation on the involvement in sexual differentiation of this protein.

**Electronic supplementary material:**

The online version of this article (doi:10.1186/s12868-016-0332-1) contains supplementary material, which is available to authorized users.

## Background

There are striking sex differences in many aspects of behaviors, not least in social behaviors such as mating and displaying of aggression [1]. Most psychiatric disorders also display sex differences in prevalence and/or symptomatology [2]. Although it is known that both sex steroids and sex chromosomes are essential for these behavioral differences as well as for sexual dimorphisms in brain anatomy and function [3], the knowledge about the proteins mediating these effects is sparse. The underlying neural circuits of social behaviors are controlled by sensory cues as well as by physiological signals, including the sex steroids. In rodents, these behaviors differ quantitatively as well as qualitatively between males and females, and rely on activity in several sexually dimorphic regions, e.g. in the amygdala and hypothalamus. In short, the medial amygdala neurons receive pheromonal input and subsequently provide afferents to specific hypothalamic nuclei [1, 4, 5], which execute social behaviors, e.g., aggression, parental behaviors and mating behaviors.

One of the most important factors responsible for sexual differentiation of the brain is testosterone. By acting during critical periods of neural development, testosterone and its metabolites cause male and female brains to develop differently [6–8]. In males the level of plasma testosterone peaks during three different time points; in utero, shortly after birth and during puberty [8]. The effects of testosterone are mediated by androgen receptors, and after aromatization to 17-β-estradiol, by the estrogen receptors (ER-α or ER-β). In rodents, estrogens through the ERs are essential for establishing male sexual and territorial behaviors whereas testosterone through the AR rather modulates the extent of these behavioral displays [1, 8]. These steroid receptors are ligand-activated transcription factors, and through the influence on the gene transcription they have the capability to regulate the expression levels of many different proteins. In addition, the sex steroids can bind to membrane bound receptors and thereby activate signal transduction pathways associated with changes in cell physiology [9].

Transcriptional effects during development are believed to be mainly responsible for sex differences in respect to neuronal numbers by differentially inducing apoptosis in the sexes [10–15], while other important sex differences regarding neuronal morphology, such as dendritic spines, dendrite length and the number of synapses are likely modulated both during development as well as later in life [11, 16–20]. Moreover, both neuroimmunological [8] and epigenetic processes [3, 21, 22] are thought to contribute to sex differentiation of these neural circuits.

To understand the origin of sexually dimorphic behaviors it is crucial to identify the proteins, which are responsible for the above mentioned structural and functional differences, and these could be assumed to be expressed differentially in males and females. Sex differences were detected already in early brain expression studies of single genes and proteins and many were due to sex steroid actions [23–25]. Later on, genome-wide studies revealed additional mRNA transcripts regulated by sex [26–33], and/or sex steroids [22, 34–37]. However, approaches such as micro-array technologies allowing large-scale analysis of gene/mRNA expression have limitations. For example, post-transcriptional events, such as alternative mRNA splicing, post-translational protein modifications, as well as regulatory mechanisms of protein translation, all change the diversity and amount of products that can be synthesized from a fixed number of genes, but are not detected on the mRNA level analysis. Regarding mRNA splicing, both sex-specific and lineage-specific alternative splicing has been shown in primates [38]. According to one study the mRNA levels explain around 40% of the variability in protein levels [39]. In contrast to mRNA-based expression analysis, proteomics has the advantage of studying proteins—the functional molecules of the cell [40]. Still, few studies have been performed concerning sex differences in protein expression on large or proteome-wide scale of the mammalian brain [41–44].

The aim of the present study was to identify proteins of importance for the sexual differentiation of amygdala and hypothalamus during early development. The masculinizing and defeminizing effects of pre- and neonatal testosterone are crucial for the sexual differentiation of the brain through actions mediated by the androgen receptor and the ERs. Therefore, we compared protein expression, using an explorative proteomics approach, in amygdala/hypothalamus samples from newborn females, males and male mice lacking androgen receptors specifically in the brain (AR^NesDel^), at a timepoint when the sex steroid receptors are known to be robustly expressed (i.e. at postnatal day 8, P8) [45]. Since the synaptic connectivity differs between the sexes and probably partly explains sexual dimorphisms in behaviors, one specific focus of our study was to explore if levels of synaptic proteins differ between the sexes.

## Methods

### Animals

In this experiment 8-day-old (P8) mice were used (n = 24). The mice were either AR^flox/y^ males (n = 6), AR^flox/−^ females (n = 6), AR^NesDel^ males (n = 6), or AR^NesCre^ males (n = 6). Generation of AR^NesDel^ has been described in detail elsewhere [46]. Briefly, mice expressing CRE driven by the neuronal *Nestin* promoter (Jackson laboratory, strain #003771) were mated with female mice with *LoxP* sites [47] flanking the second exon of the androgen receptor gene. Genotypes were confirmed with PCR. Mice were kept in the breeding cage until sacrificed at P8. Breeding cages were held in a conventional animal facility with 12 h light/12 h dark cycle, lights on at 6.00 a.m., and given ad libitum access to food and water.

### Brain tissue

After decapitation of the mice the brains were removed, immediately frozen in liquid nitrogen and stored at −80 °C until subsequent use. Dissection of the hypothalamus and amygdala was performed by placing the brains with the ventral side facing up in a Young Mouse Brain Coronal Brain Slicer (matrix) from Zivic Instruments (http://www.zivicinstruments.com). Four vertical cuts spaced 1 mm apart were made, using razor blades. The first cut was made just rostrally of the cerebellum, and together with the three following cuts sections between 3.39 mm (#17) and 6.27 mm (#41), according to the P6 series in the Atlas of the developing mouse brain by Paxinos et al. [48], were generated. The hypothalamus and amygdala were subsequently collected from these sections, using razor blades. The tissue encompassing the hypothalamus and amygdala was transferred to Eppendorf tubes at −20 °C and then stored at −80 °C until the homogenization process (see below).

### Global quantitative proteomic analyses using iTRAQ

Samples were homogenized in lysis buffer [2% SDS in 50 mM triethylammonium bicarbonate (TEAB)]. Each sample (100 µg) and a reference pool (100 µg) containing equal amounts of all samples were trypsin digested using filter-aided sample preparation (FASP) [49] followed by iTRAQ reagent labeling (Applied Biosystems) into nine 4-plexed sets. The iTRAQ sets were fractionated by Strong Cation Exchange Chromatography (ÄKTA-system, Amersham-Pharmacia) on a PolySULFOETHYL A™ column (100 × 2.1 mm, 5 µm 300 Å, PolyLC inc.) with a gradient from 25 to 500 mM ammonium formate (pH 2.8) in 20% ACN over 40 min. Twenty fractions from each set were desalted using PepClean C18 spin columns according to manufacturer’s instructions (Thermo Fisher Scientific), dried down and reconstituted with 15 µL of 0.1% formic acid in 3% acetonitrile.

The MS and MS/MS were performed on an LTQ Orbitrap Velos mass spectrometer interfaced to an Easy-nLC II (Thermo Fisher Scientific) in a data-dependent mode. Peptides were separated at 200 nL/min using an in-house constructed analytical column (200 × 0.075 mm I.D., 3 μm C18, Dr. Maisch, Germany) with a gradient from 5 to 80% acetonitrile in 0.2% formic acid over 90 min. MS scans were performed at m/z range 400–1800 and the ten most abundant peptides were selected simultaneous for MS/MS-fragmentation by HCD for identification and quantification.

MS-raw data for each iTRAQ-set were merged for relative quantification and identification using Proteome Discoverer version 1.3 (Thermo Fisher Scientific). Mascot (Matrix Science) were selected for the database search with the parameters *Mus musculus* Swissprot Database version 2.3 (Swiss Institute of Bioinformatics, Switzerland), 10 ppm peptide tolerance, 100 mmu MS/MS tolerance, one missed cleavages, variable methionine oxidation and cysteine alkylation modifications, fixed iTRAQ-label modifications of N-terminals and lysines at 1% False Discovery Rate. For quantification, the ratios of the iTRAQ-reporter ion intensities in MS/MS spectra were used. Only unique peptides were considered for quantitation.

Welch’s t test was used to compare differences in protein expression between male and female mice, and subsequently between male and AR^NesDel^ male mice. Because of the explorative design in this study, a nominal p value level of ≤0.05 was used. For all proteins with a p value of ≤0.05 in the t test, the fold change (mean male ratio versus mean female ratio, or mean male ratio versus mean AR^NesDel^ male ratio) was calculated. Proteins with missing expression values for more than three samples were not included in the analyses. Furthermore, the variance (SD/mean) in percent was calculated for each protein, to make sure that this would not exceed the percent of up- or down-regulation for a specific protein.

The following proteins were found to be differentially expressed in both males versus AR^NesDel^ males and males versus AR^NesCre^ males: basal cell adhesion molecule, Ras-related protein Rap-1b, microtubule-associated serine/threonine-protein kinase 3 and lambda-crystallin homolog. This means that we cannot exclude that Nes-Cre by itself may affect the expression levels of these proteins, and, subsequently, they were excluded from the list of proteins differentially expressed between males and AR^NesDel^ males.

### Targeted quantitative proteomic analyses using parallel reaction monitoring (PRM)

The selected peptides (JPT peptide Technologies GmbH, Berlin, Germany) were unique to the seven different proteins of interest (presented in Table 1) and contained a heavy lysine (^13^C6, ^15^N2) or arginine (^13^C6, ^15^N4). Initially calibration curves were prepared by digestion of a representative tissue extract together with the labeled peptide as described above, resulting in samples with a final concentration of 1 µg/μL total protein containing 3.1, 6.3, 12.5, 25, 50 or 100 fmol/μL of the labeled peptide. MS analyses and evaluation were performed as described below. The MS response was found to be linear within this concentration range.

**Table 1:** Results from iTRAQ analyses for the proteins included in targeted proteomics

Proteins	Uniprot accession nr	Fold change^a^	p value^b^
Scaffolders and adaptors			
Neurobeachin	Q9EPN1	1.09	0.022
G-proteins and modulators			
Neuromodulin	P06837	−1.1	0.002
Septin-7	O55131	−1.1	0.002
Cytoskeletal and cell adhesion			
Myristoylated alanine-rich C-kinase substrate	P26645	−1.22	0.007
Neurochondrin	Q9Z0E0	1.12	0.049
Transcription and translation			
General transcription factor II-I	Q9ESZ8	1.15	0.049

^a^Fold changes between males and females. Positive values represent male-biased proteins and negative values represent female-biased proteins

^b^Proteins differentially expressed (p ≤ 0.05) in males and females

The homogenized amygdala/hypothalamus samples (30 µg each) from the groups of males, females and AR^NesDel^ males (the same samples that were used in the iTRAQ-analyses) were digested with trypsin as above, except that the labeled peptides were added together with the trypsin. The samples were spiked with the labeled peptides resulting in a concentration close to the one determined for the endogenous peptide in the sample analyzed in the dilution series. The final samples for injection contained the labeled peptides in concentrations of 6.3, 12.5, 25, 50 or 100 fmol/µL depending on peptide.

The PRM analyses were performed using a Q-Exactive mass spectrometer interfaced to an Easy-nLC II (Thermo Fisher Scientific). Peptides were separated as above except that a gradient from 5 to 80% acetonitrile in 0.2% formic acid over 40 min was used. For the PRM method, an orbitrap resolution of 35,000 and a quadrupole isolation window of 2 Th were used. For MARCKS the sequence of the peptide for quantitative analysis was AEDGAAPSPSSETPK. The precursor ions of the labeled peptide (*m/z* 726.3492) and endogenous peptide (*m/z* 722.3335) were targeted at their 2+ charge in ±4 min elution time window and fragmented by HCD.

MS raw data files for each sample were database searched and the results were imported into Skyline. The most intense fragments of the corresponding pair of endogenous [*m/z* 1000.4946 (y10), 929.4575 (y9), 704.3727 (y7)] and labeled [*m/z* 1008.4796 (y10), 937.4717 (y9), 753.3869 (y7)] peptides were selected to be used for quantification of MARCKS. For the selected fragments a minimal degree of interference occurring during the elution of the peptides were confirmed. To determine the concentration of the endogenous peptide from the concentration of the labeled peptide (50 fmol/µL, quantification based on single point measurement concept) the ratio to standard (endogenous/labeled peptide) × concentration labeled peptide was used. For comparison of protein expression between groups Welch’s t test was used. One male study sample was excluded from the PRM study due to interference in the MS-analysis that obstructed the quantification.

## Results

In the present study, we were able to identify a total of 2998 proteins in hypothalamus and amygdala of neonatal male, female and AR^NesDel^ male mice. After excluding proteins lacking expression values for more than three samples, 2273 proteins were compared between the sexes and 2293 proteins were compared between AR^NesDel^ males and male controls. Based on a nominal p value level of ≤0.05, 173 proteins were differentially expressed in males and females (Additional file 1: Table S1), whereas 38 proteins varied between males and AR^NesDel^ males (Additional file 2: Table S2). Further analyses revealed that 65 of the sexually dimorphic proteins and 13 of the proteins differentially expressed in males and AR^NesDel^ males showed a fold change ≥1.1 (male-biased in males versus females or males versus AR^NesDel^) or ≤−1.1 (female-biased in males versus females and AR^NesDel^-biased in males versus AR^NesDel^). The overlap between the proteins differentially expressed in males vs. females and in males vs. AR^NesDel^ males was found to be small; only the proteins Actin (alpha cardiac muscle 1) and Protein CutA were found to be altered in both comparisons.

**Fig. 1:**
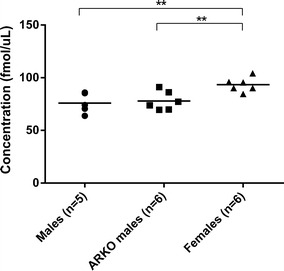
Protein expression levels of MARCKS based on results from targeted proteomics. Due to interference in the MS-analysis that obstructed the quantification, one male sample was excluded from this experiment. Comparisons of protein expression levels of MARCKS between male (n = 5) and female (n = 6) mice, and subsequently between female and AR^NesDel^ male (n = 6) mice, at postnatal day 8, show a significantly higher expression in females compared to both males and AR^NesDel^ males, **p ≤ 0.01. The difference between males and AR^NesDel^ males was non-significant (p = 0.72). The comparisons were performed using Welch’s t test

Since one specific focus of our study was to explore differences in proteins of crucial importance for the synaptic physiology, seven proteins, among the ones with a fold change of ≥1.1 or ≤−1.1 and related to synaptic connectivity, were chosen for verification analyses with targeted proteomics (Table 1). The relation with synaptic connectivity relied on the previous literature, as well as expert curated gene sets involving genes active in the pre- and post-synapse [50, 51], found at http://ctglab.nl/software/genesets. The result of the targeted proteomics analysis verified that one of the proteins, MARCKS (myristoylated alanine rich C kinase substrate), was differentially expressed in males and females. The concentrations of the MARCKS were significantly changed in the male and AR^NesDel^ male groups compared with the female group (p = 0.01 for both comparisons, see Fig. 1). In line with our results from the iTRAQ analyses, no significant difference in MARCKS expression levels was found between the male and AR^NesDel^ male groups.

## Discussion

To understand more about the molecular mechanisms underlying sexual dimorphisms in behavior and brain function, we here compared protein expression in hypothalamus/amygdala from neonatal males and females. Our explorative proteomic approach allowed us to investigate differences in approximately 2300 of the most highly-expressed proteins in our dissected tissues. No large changes in any specific protein were revealed, but many proteins (>170) displayed small expression differences between the sexes (fold changes of less than 1.34 or more than −1.31). Importantly, considering that approximately 2300 proteins were compared between groups about 115 proteins of these should be expected to be false positives at the selection threshold p = 0.05. Interestingly however, the MARCKS protein was verified as differentially expressed between the sexes using targeted proteomics.

There is consistent evidence for sex differences in morphology and synaptic connectivity in the hypothalamus, the amygdala as well as in many other brain regions [11]. Therefore it is interesting that the synaptic protein MARCKS was identified as sexually dimorphic with both our proteomic approaches, iTRAQ and targeted proteomics. MARKCS is a cellular substrate for protein kinase C and is highly expressed in the brain, especially during development [52, 53]. MARCKS has been shown to be of importance for synaptic signaling [54] and plasticity [55] and is necessary for normal mouse brain development [52]. Previous studies have mainly focused on the role of this protein in processes related to memory [56, 57] and learning [53]. The present study is the first to report sex differences in brain protein levels of MARCKS and this result may provide knowledge regarding the molecular mechanisms involved in the well-established sex differences with respect to, for example, dendritic spines, dendrite length and the number of synapses [11]. Future studies should confirm our finding of sexually dimorphic expression of MARCKS in neonates, but also extend the investigations to other developmental stages and more specific nuclei of the limbic system.

The subtle magnitudes of the sex differences revealed in our study are very much in line with previous exploratory studies of sexually dimorphic gene expression [27, 33, 58–60]. For example, in a microarray study by Yang et al. [27] 613 of 4508 brain expressed genes differed by >onefold between the sexes, and 37 of those 613 genes displayed a fold difference of >1.2. Likewise, also in a recent study of postmortem human brains from men and women only subtle gene expression differences were detected [61]. Small expression differences between the sexes were also confirmed on the protein level in a recent study of approximately 100 proteins in several brain regions [41] showing that only 2 of the 51 sexually dimorphic proteins differed by >30%. As previously suggested [60], this may indicate that differences between the sexes in brain function and behavior may arise from combinatorial effects of a large number of proteins. Moreover, recent studies indicate that the sexually dimorphic and hormone regulated gene expression may mainly be restricted to specific neurons of certain brain nuclei [22, 37, 59, 62]. Hence, our choice of tissue dissection comprising the many nuclei and neuronal types of hypothalamus and amygdala may have compromised the possibility to reveal larger differences in protein expression. Furthermore, as the present study did not include samples from brain areas not expressing sex steroid receptors we cannot evaluate if the subtle sex differences extend to areas of the brain not influenced by gonadal hormones. Thus, inclusion of such control samples in future large-scale studies is warranted. Taken together, future studies should attempt to investigate protein expression in several specifically dissected brain regions in large sets of samples.

To our knowledge, no previous studies have compared expression differences between AR^NesDel^ mice and controls on a genome-wide or proteome-wide basis. However, a number of studies have investigated the effects of sex steroid treatment on gene expression in normal mice [22, 34–37, 63]. By using the AR^NesDel^ mouse line, known to have no or low expression of androgen receptors in the brain already during embryonal development [45, 46, 64–66], it was possible to specifically investigate if differentially expressed proteins were regulated by the androgen receptor. Surprisingly, only small differences of less than 40 proteins were seen between males expressing and not expressing androgen receptors in the brain, indicating that the influence of the androgen receptor on protein levels in the investigated brain areas at the age of P8 is limited. Juntti et al. [45] showed that the androgen receptor is expressed from P7, but our data may indicate that its effects on protein expression occur later on.

In the present study we used iTRAQ (isobaric tags for relative and absolute quantitation) for unbiased proteomics analysis that allows simultaneous relative quantification of proteins in multiple samples. The technique offers reduced systematic error and increased efficiency leading to higher sensitivity [67]. For absolute quantification of the seven selected proteins, targeted quantitative proteomics using parallel reaction monitoring (PRM) was performed. Isotopically labelled peptides containing unique sequences for the selected proteins were spiked at known concentrations into the samples allowing absolute quantification of the peptides and their corresponding proteins. During the PRM analysis the fragment ions of the endogenous and its isotopically labelled peptides were monitored simultaneous at high resolution, which reduces interferences and significantly enhances the selectivity of the method. Using PRM, which also is a more sensitive method compared to iTRAQ, for investigations of proteins differentially expressed in the global proteomic analysis, showed that only fold changes over 20% could be verified. This result suggests that fold changes around 10% detected in the iTRAQ analysis were difficult to verify due to our relatively small sample size. Furthermore, although iTRAQ detects a large number of proteins (up to about 3000 in the tissues investigated in this study), there are most likely relevant proteins which are affected by sex or androgen receptors but which remain undetected by using this approach.

## Conclusions

Our results may suggest that a large number of proteins could be involved in the sexual differentiation of the brain, since we observed many subtle but no large sex differences for single proteins. Future analyses using more sensitive detection methods of samples from specific brain nuclei from large number of samples may however modify this view. The importance of our finding of a sexual dimorphic expression of MARCKS for sexual differentiation of the brain needs to be clarified by future research.

## Additional files

**Additional file 1: Table S1.** Differentially expressed proteins in amygdala and hypothalamus of 8-day-old male and female mice.

**Additional file 2: Table S2.** Differentially expressed proteins in amygdala and hypothalamus of 8-day-old male and AR^NesDel^ male mice.

## References

[1] Manoli DS, Fan P, Fraser EJ, Shah NM (2013). Neural control of sexually dimorphic behaviors. Curr Opin Neurobiol.

[2] Eaton NR, Keyes KM, Krueger RF, Balsis S, Skodol AE, Markon KE (2012). An invariant dimensional liability model of gender differences in mental disorder prevalence: evidence from a national sample. J Abnorm Psychol.

[3] McCarthy MM, Pickett LA, VanRyzin JW, Kight KE (2015). Surprising origins of sex differences in the brain. Horm Behav.

[4] Canteras NS, Simerly RB, Swanson LW (1995). Organization of projections from the medial nucleus of the amygdala: a PHAL study in the rat. J Comp Neurol.

[5] Dulac C, Wagner S (2006). Genetic analysis of brain circuits underlying pheromone signaling. Annu Rev Genet.

[6] De Bellis MD, Keshavan MS, Beers SR, Hall J, Frustaci K, Masalehdan A (2001). Sex differences in brain maturation during childhood and adolescence. Cereb Cortex.

[7] Hutchison JB (1997). Gender-specific steroid metabolism in neural differentiation. Cell Mol Neurobiol.

[8] Lenz KM, Nugent BM, McCarthy MM (2012). Sexual differentiation of the rodent brain: dogma and beyond. Front Neurosci.

[9] Woolley CS (2007). Acute effects of estrogen on neuronal physiology. Annu Rev Pharmacol Toxicol.

[10] Forger NG, Rosen GJ, Waters EM, Jacob D, Simerly RB, de Vries GJ (2004). Deletion of Bax eliminates sex differences in the mouse forebrain. Proc Natl Acad Sci USA.

[11] McCarthy MM, Arnold AP (2011). Reframing sexual differentiation of the brain. Nat Neurosci.

[12] Krishnan S, Intlekofer KA, Aggison LK, Petersen SL (2009). Central role of TRAF-interacting protein in a new model of brain sexual differentiation. Proc Natl Acad Sci USA.

[13] Waters EM, Simerly RB (2009). Estrogen induces caspase-dependent cell death during hypothalamic development. J Neurosci.

[14] Gotsiridze T, Kang N, Jacob D, Forger NG (2007). Development of sex differences in the principal nucleus of the bed nucleus of the stria terminalis of mice: role of Bax-dependent cell death. Dev Neurobiol.

[15] Gilmore RF, Varnum MM, Forger NG (2012). Effects of blocking developmental cell death on sexually dimorphic calbindin cell groups in the preoptic area and bed nucleus of the stria terminalis. Biol Sex Differ.

[16] Amateau SK, McCarthy MM (2004). Induction of PGE(2) by estradiol mediates developmental masculinization of sex behavior. Nat Neurosci.

[17] Mong JA, Glaser E, McCarthy MM (1999). Gonadal steroids promote glial differentiation and alter neuronal morphology in the developing hypothalamus in a regionally specific manner. J Neurosci.

[18] Todd BJ, Schwarz JM, Mong JA, McCarthy MM (2007). Glutamate AMPA/kainate receptors, not GABA(A) receptors, mediate estradiol-induced sex differences in the hypothalamus. Dev Neurobiol.

[19] Cooke BM (2006). Steroid-dependent plasticity in the medial amygdala. Neuroscience.

[20] Cooke BM, Woolley CS (2005). Gonadal hormone modulation of dendrites in the mammalian CNS. J Neurobiol.

[21] McCarthy MM, Auger AP, Bale TL, De Vries GJ, Dunn GA, Forger NG (2009). The epigenetics of sex differences in the brain. J Neurosci.

[22] Ghahramani NM, Ngun TC, Chen PY, Tian Y, Krishnan S, Muir S (2014). The effects of perinatal testosterone exposure on the DNA methylome of the mouse brain are late-emerging. Biol Sex Differ.

[23] Lauber AH, Mobbs CV, Muramatsu M, Pfaff DW (1991). Estrogen receptor messenger RNA expression in rat hypothalamus as a function of genetic sex and estrogen dose. Endocrinology.

[24] Lustig RH, Sudol M, Pfaff DW, Federoff HJ (1991). Estrogenic regulation and sex dimorphism of growth-associated protein 43 kDa (GAP-43) messenger RNA in the rat. Brain Res Mol Brain Res.

[25] Olazabal UE, Pfaff DW, Mobbs CV (1992). Sex differences in the regulation of heat shock protein 70 kDa and 90 kDa in the rat ventromedial hypothalamus by estrogen. Brain Res.

[26] Rinn JL, Rozowsky JS, Laurenzi IJ, Petersen PH, Zou K, Zhong W (2004). Major molecular differences between mammalian sexes are involved in drug metabolism and renal function. Dev Cell.

[27] Yang X, Schadt EE, Wang S, Wang H, Arnold AP, Ingram-Drake L (2006). Tissue-specific expression and regulation of sexually dimorphic genes in mice. Genome Res.

[28] Vawter MP, Evans S, Choudary P, Tomita H, Meador-Woodruff J, Molnar M (2004). Gender-specific gene expression in post-mortem human brain: localization to sex chromosomes. Neuropsychopharmacology.

[29] Weickert CS, Elashoff M, Richards AB, Sinclair D, Bahn S, Paabo S (2009). Transcriptome analysis of male–female differences in prefrontal cortical development. Mol Psychiatry.

[30] Reinius B, Jazin E (2009). Prenatal sex differences in the human brain. Mol Psychiatry.

[31] Dewing P, Shi T, Horvath S, Vilain E (2003). Sexually dimorphic gene expression in mouse brain precedes gonadal differentiation. Brain Res Mol Brain Res.

[32] Itoh Y, Arnold AP (2015). Are females more variable than males in gene expression? Meta-analysis of microarray datasets. Biol Sex Differ.

[33] Reinius B, Shi C, Hengshuo L, Sandhu KS, Radomska KJ, Rosen GD (2010). Female-biased expression of long non-coding RNAs in domains that escape X-inactivation in mouse. BMC Genom.

[34] Peterson MP, Rosvall KA, Choi JH, Ziegenfus C, Tang H, Colbourne JK (2013). Testosterone affects neural gene expression differently in male and female juncos: a role for hormones in mediating sexual dimorphism and conflict. PLoS ONE.

[35] Sakakibara M, Uenoyama Y, Minabe S, Watanabe Y, Deura C, Nakamura S (2013). Microarray analysis of perinatal-estrogen-induced changes in gene expression related to brain sexual differentiation in mice. PLoS ONE.

[36] Wada-Kiyama Y, Suzuki C, Hamada T, Rai D, Kiyama R, Kaneda M (2013). Estrogen-induced cell signaling in the sexually dimorphic nucleus of the rat preoptic area: potential involvement of cofilin in actin dynamics for cell migration. Biochem Biophys Res Commun.

[37] Lichtensteiger W, Bassetti-Gaille C, Faass O, Axelstad M, Boberg J, Christiansen S (2015). Differential gene expression patterns in developing sexually dimorphic rat brain regions exposed to antiandrogenic, estrogenic, or complex endocrine disruptor mixtures: glutamatergic synapses as target. Endocrinology.

[38] Blekhman R, Marioni JC, Zumbo P, Stephens M, Gilad Y (2010). Sex-specific and lineage-specific alternative splicing in primates. Genome Res.

[39] Schwanhausser B, Busse D, Li N, Dittmar G, Schuchhardt J, Wolf J (2011). Global quantification of mammalian gene expression control. Nature.

[40] Taurines R, Dudley E, Grassl J, Warnke A, Gerlach M, Coogan AN (2011). Proteomic research in psychiatry. J Psychopharmacol.

[41] Block A, Ahmed MM, Dhanasekaran AR, Tong S, Gardiner KJ (2015). Sex differences in protein expression in the mouse brain and their perturbations in a model of Down syndrome. Biol Sex Differ.

[42] Shi L, Du X, Zhou H, Tao C, Liu Y, Meng F (2014). Cumulative effects of the ApoE genotype and gender on the synaptic proteome and oxidative stress in the mouse brain. Int J Neuropsychopharmacol.

[43] Di Domenico F, Casalena G, Jia J, Sultana R, Barone E, Cai J (2012). Sex differences in brain proteomes of neuron-specific STAT3-null mice after cerebral ischemia/reperfusion. J Neurochem.

[44] Di Domenico F, Casalena G, Sultana R, Cai J, Pierce WM, Perluigi M (2010). Involvement of Stat3 in mouse brain development and sexual dimorphism: a proteomics approach. Brain Res.

[45] Juntti SA, Tollkuhn J, Wu MV, Fraser EJ, Soderborg T, Tan S (2010). The androgen receptor governs the execution, but not programming, of male sexual and territorial behaviors. Neuron.

[46] Raskin K, de Gendt K, Duittoz A, Liere P, Verhoeven G, Tronche F (2009). Conditional inactivation of androgen receptor gene in the nervous system: effects on male behavioral and neuroendocrine responses. J Neurosci.

[47] De Gendt K, Swinnen JV, Saunders PT, Schoonjans L, Dewerchin M, Devos A (2004). A sertoli cell-selective knockout of the androgen receptor causes spermatogenic arrest in meiosis. Proc Natl Acad Sci USA.

[48] Paxinos G, Halliday G, Watson C, Koutcherov Y, Wang H (2007). Atlas of the developing mouse brain.

[49] Wisniewski JR, Zougman A, Nagaraj N, Mann M (2009). Universal sample preparation method for proteome analysis. Nat Methods.

[50] Ruano D, Abecasis GR, Glaser B, Lips ES, Cornelisse LN, de Jong AP (2010). Functional gene group analysis reveals a role of synaptic heterotrimeric G proteins in cognitive ability. Am J Hum Genet.

[51] Lips ES, Cornelisse LN, Toonen RF, Min JL, Hultman CM, International Schizophrenia C (2012). Functional gene group analysis identifies synaptic gene groups as risk factor for schizophrenia. Mol Psychiatry.

[52] Stumpo DJ, Bock CB, Tuttle JS, Blackshear PJ (1995). MARCKS deficiency in mice leads to abnormal brain development and perinatal death. Proc Natl Acad Sci USA.

[53] McNamara RK, Stumpo DJ, Morel LM, Lewis MH, Wakeland EK, Blackshear PJ (1998). Effect of reduced myristoylated alanine-rich C kinase substrate expression on hippocampal mossy fiber development and spatial learning in mutant mice: transgenic rescue and interactions with gene background. Proc Natl Acad Sci USA.

[54] Mosevitsky MI (2005). Nerve ending “signal” proteins GAP-43, MARCKS, and BASP1. Int Rev Cytol.

[55] Calabrese B, Halpain S (2005). Essential role for the PKC target MARCKS in maintaining dendritic spine morphology. Neuron.

[56] Solomonia RO, Morgan K, Kotorashvili A, McCabe BJ, Jackson AP, Horn G (2003). Analysis of differential gene expression supports a role for amyloid precursor protein and a protein kinase C substrate (MARCKS) in long-term memory. Eur J Neurosci.

[57] Hussain RJ, Stumpo DJ, Blackshear PJ, Lenox RH, Abel T, McNamara RK (2006). Myristoylated alanine rich C kinase substrate (MARCKS) heterozygous mutant mice exhibit deficits in hippocampal mossy fiber-CA3 long-term potentiation. Hippocampus.

[58] Armoskus C, Moreira D, Bollinger K, Jimenez O, Taniguchi S, Tsai HW (2014). Identification of sexually dimorphic genes in the neonatal mouse cortex and hippocampus. Brain Res.

[59] Xu X, Coats JK, Yang CF, Wang A, Ahmed OM, Alvarado M (2012). Modular genetic control of sexually dimorphic behaviors. Cell.

[60] Mozhui K, Lu L, Armstrong WE, Williams RW (2012). Sex-specific modulation of gene expression networks in murine hypothalamus. Front Neurosci.

[61] Werling DM, Parikshak NN, Geschwind DH (2016). Gene expression in human brain implicates sexually dimorphic pathways in autism spectrum disorders. Nat Commun.

[62] Yang CF, Shah NM (2014). Representing sex in the brain, one module at a time. Neuron.

[63] Yin W, Maguire SM, Pham B, Garcia AN, Dang NV, Liang J (2015). Testing the critical window hypothesis of timing and duration of estradiol treatment on hypothalamic gene networks in reproductively mature and aging female rats. Endocrinology.

[64] Karlsson SA, Studer E, Kettunen P, Westberg L (2016). Neural androgen receptors modulate gene expression and social recognition but not social investigation. Front Behav Neurosci.

[65] Chen CV, Brummet JL, Jordan CL, Breedlove SM (2016). Down, but not out: partial elimination of androgen receptors in the male mouse brain does not affect androgenic regulation of anxiety or HPA activity. Endocrinology.

[66] Tronche F, Kellendonk C, Kretz O, Gass P, Anlag K, Orban PC (1999). Disruption of the glucocorticoid receptor gene in the nervous system results in reduced anxiety. Nat Genet.

[67] Ross PL, Huang YN, Marchese JN, Williamson B, Parker K, Hattan S (2004). Multiplexed protein quantitation in *Saccharomyces cerevisiae* using amine-reactive isobaric tagging reagents. Mol Cell Proteom.

