# Supplementary material for: Proteomic analyses of limbic regions in neonatal male, female and androgen receptor knockout mice
Source: BMC Neurosci. 2017 Jan 5;18:9. doi: 10.1186/s12868-016-0332-1 (PMC5217640; doi:10.1186/s12868-016-0332-1)
Supplement: Supplementary file 1 — Additional file 1: Table S1. Differentially expressed proteins in amygdala and hypothalamus of 8-day-old male and female mice. [file 12868_2016_332_MOESM1_ESM.docx]

**Table S1.** Differentially expressed proteins in amygdala and hypothalamus

of 8-day-old male and female mice.

| **Proteins** | **Gene**  **symbol** | **Uniprot Accession Nr** | **Fold change^a^** | **p-value^b^** |
| --- | --- | --- | --- | --- |
| *Channels and Receptors* |  |  |  |  |
| Phospholemman | *Fxyd1* | Q9Z239 | -1.29 | 0.025 |
| Opioid growth factor receptor | *Ogfr* | Q99PG2 | -1.09 | 0.031 |
|  |  |  |  |  |
| *Scaffolders and Adaptors* |  |  |  |  |
| Na(+)/H(+) exchange regulatory cofactor NHE-RF1 | *Slc9a3r1* | P70441 | -1.18 | 0.025 |
| Annexin A2 | *Anxa2* | P07356 | -1.16 | 0.040 |
| 14-3-3 protein eta | *Ywhah* | P68510 | -1.12 | 0.002 |
| 14-3-3 protein gamma | *Ywhag* | P61982 | -1.10 | 0.004 |
| S-phase kinase-associated protein 1 | *Skp1* | Q9WTX5 | -1.09 | 0.004 |
| 14-3-3 protein beta/alpha | *Ywhab* | Q9CQV8 | -1.06 | 0.045 |
| 14-3-3 protein epsilon | *Ywhae* | P62259 | -1.04 | 0.016 |
| 14-3-3 protein zeta/delta | *Ywhaz* | P63101 | -1.04 | 0.011 |
| Stress-induced-phosphoprotein 1 | *Stip1* | Q60864 | -1.02 | 0.034 |
| Peripheral plasma membrane protein CASK | *Cask* | O70589 | 1.03 | 0.041 |
| Epidermal growth factor receptor substrate 15-like 1 | *Eps15l1* | Q60902 | 1.06 | 0.015 |
| Adapter molecule crk | *Crk* | Q64010 | 1.09 | 0.038 |
| Neurobeachin | *Nbea* | Q9EPN1 | 1.09 | 0.022 |
| MAGUK p55 subfamily member 6 | *Mpp6* | Q9JLB0 | 1.12 | 0.042 |
| C-Jun-amino-terminal kinase-interacting protein 1 | *Mapk8ip1* | Q9WVI9 | 1.15 | 0.010 |
| Disks large-associated protein 4 | *Dlgap4* | B1AZP2 | 1.16 | 0.028 |
|  |  |  |  |  |
| *Signalling Molecules and Enzymes* |  |  |  |  |
| Arginyl-tRNA--protein transferase 1 | *Ate1* | Q9Z2A5 | -1.20 | 0.017 |
| Alkaline phosphatase, tissue-nonspecific isozyme | *Alpl* | P09242 | -1.15 | 0.015 |
| Nardilysin | *Nrd1* | Q8BHG1 | -1.15 | 0.009 |
| Cullin-4B | *Cul4b* | A2A432 | -1.14 | 0.027 |
| Alpha-1,3-mannosyltransferase ALG2 | *Alg2* | Q9DBE8 | -1.12 | 0.004 |
| Small glutamine-rich tetratricopeptide repeat-  containing protein alpha | *Sgta* | Q8BJU0 | -1.11 | 0.004 |
| Brain acid soluble protein 1 | *Basp1* | Q91XV3 | -1.09 | 0.013 |
| Regulator of G-protein signaling 10 | *Rgs10* | Q9CQE5 | -1.09 | 0.027 |
| ATP synthase subunit b, mitochondrial | *Atp5f1* | Q9CQQ7 | -1.09 | 0.025 |
| Cell cycle exit and neuronal differentiation protein 1 | *Cend1* | Q9JKC6 | -1.08 | 0.033 |
| Signal peptidase complex subunit 2 | *Spcs2* | Q9CYN2 | -1.08 | 0.048 |
| Anaphase-promoting complex subunit 1 | *Anapc1* | P53995 | -1.07 | 0.049 |
| Proteasome subunit alpha type-1 | *Psma1* | Q9R1P4 | -1.07 | 0.011 |
| Proteasome subunit alpha type-7 | *Psma7* | Q9Z2U0 | -1.07 | 0.029 |
| Calpain-2 catalytic subunit | *Capn2* | O08529 | -1.07 | 0.026 |
| Peroxiredoxin-2 | *Prdx2* | Q61171 | -1.06 | 0.014 |
| 78 kDa glucose-regulated protein | *Hspa5* | P20029 | -1.06 | 0.008 |
| 10-formyltetrahydrofolate dehydrogenase | *Aldh1l1* | Q8R0Y6 | -1.06 | 0.009 |
| Dolichyl-diphosphooligosaccharide-  protein glycosyltransferase subunit 2 | *Rpn2* | Q9DBG6 | -1.06 | 0.041 |
| Heat shock protein HSP 90-alpha | *Hsp90aa1* | P07901 | -1.05 | 0.002 |
| UMP-CMP kinase | *Cmpk1* | Q9DBP5 | -1.05 | 0.027 |
| Creatine kinase B-type | *Ckb* | Q04447 | -1.05 | 0.008 |
| Heat shock 70 kDa protein 4 | *Hspa4* | Q61316 | -1.05 | 0.003 |
| Endoplasmin | *Hsp90b1* | P08113 | -1.05 | 0.027 |
| Calreticulin | *Calr* | P14211 | -1.04 | 0.031 |
| L-lactate dehydrogenase B chain | *Ldhb* | P16125 | -1.04 | 0.035 |
| ATP synthase subunit alpha, mitochondrial | *Atp5a1* | Q03265 | -1.04 | 0.034 |
| Puromycin-sensitive aminopeptidase | *Npepps* | Q11011 | -1.02 | 0.008 |
| Aspartyl-tRNA synthetase, cytoplasmic | *Dars* | Q922B2 | 1.05 | 0.011 |
| NADH dehydrogenase [ubiquinone] 1  alpha subcomplex subunit 8 | *Ndufa8* | Q9DCJ5 | 1.05 | 0.036 |
| Methylosome subunit pICln | *Clns1a* | Q61189 | 1.07 | 0.026 |
| Bifunctional coenzyme A synthase | *Coasy* | Q9DBL7 | 1.07 | 0.010 |
| Hepatocyte growth factor-  regulated tyrosine kinase substrate | *Hgs* | Q99LI8 | 1.07 | 0.004 |
| N-alpha-acetyltransferase 35, NatC auxiliary subunit | *Naa35* | Q6PHQ8 | 1.07 | 0.012 |
| Glycerol-3-phosphate dehydrogenase 1-like protein | *Gpd1l* | Q3ULJ0 | 1.08 | 0.001 |
| Hypoxanthine-guanine phosphoribosyltransferase | *Hprt1* | P00493 | 1.08 | 0.038 |
| Acetyl-CoA acetyltransferase, cytosolic | *Acat2* | Q8CAY6 | 1.08 | 0.023 |
| Lambda-crystallin homolog | *Cryl1* | Q99KP3 | 1.09 | 0.040 |
| NADH dehydrogenase [ubiquinone]  iron-sulfur protein 7, mitochondrial | *Ndufs7* | Q9DC70 | 1.09 | 0.030 |
| Peptidyl-prolyl cis-trans isomerase NIMA-interacting 1 | *Pin1* | Q9QUR7 | 1.10 | 0.020 |
| DnaJ homolog subfamily C member 11 | *Dnajc11* | Q5U458 | 1.10 | 0.032 |
| Acylphosphatase-1 | *Acyp1* | P56376 | 1.11 | 0.033 |
| Cytoplasmic phosphatidylinositol transfer protein 1 | *Pitpnc1* | Q8K4R4 | 1.12 | 0.048 |
| NADH dehydrogenase [ubiquinone] 1  alpha subcomplex subunit 6 | *Ndufa6* | Q9CQZ5 | 1.12 | 0.013 |
| Cathepsin B | *Ctsb* | P10605 | 1.13 | 0.044 |
| Striatin | *Strn* | O55106 | 1.14 | 0.031 |
| Striatin-4 | *Strn4* | P58404 | 1.14 | 0.026 |
| Methionine aminopeptidase 1 | *Metap1* | Q8BP48 | 1.17 | 0.002 |
| Protein CutA | *Cuta* | Q9CQ89 | 1.17 | 0.018 |
| DAZ-associated protein 1 | *Dazap1* | Q9JII5 | 1.18 | 0.028 |
| Cullin-5 | *Cul5* | Q9D5V5 | 1.20 | 0.041 |
| NEDD8 ultimate buster 1 | *Nub1* | P54729 | 1.23 | 0.010 |
| NADH dehydrogenase [ubiquinone] 1  beta subcomplex subunit 7 | *Ndufb7* | Q9CR61 | 1.31 | 0.049 |
|  |  |  |  |  |
| *Ser/Thr Kinases* |  |  |  |  |
| cAMP-dependent protein kinase type II-  alpha regulatory subunit | *Prkar2a* | P12367 | -1.13 | 0.009 |
| Protein kinase C beta type | *Prkcb* | P68404 | 1.14 | 0.024 |
| Traf2 and NCK-interacting protein kinase | *Tnik* | P83510 | 1.18 | 0.044 |
| Protein kinase C gamma type | *Prkcg* | P63318 | 1.24 | 0.031 |
|  |  |  |  |  |
| *Protein Phosphatases* |  |  |  |  |
| Protein phosphatase 1E | *Ppm1e* | Q80TL0 |  | 0.019 |
|  |  |  |  |  |
| *G-proteins and Modulators* |  |  |  |  |
| Synembryn-A | *Ric8a* | Q3TIR3 | -1.12 | 0.020 |
| Neuromodulin | *Gap43* | P06837 | -1.10 | 0.002 |
| Septin-7 | *07-sep* | O55131 | -1.10 | 0.002 |
| Ras-related protein Rab-10 | *Rab10* | P61027 | -1.09 | 0.046 |
| Rho GDP-dissociation inhibitor 1 | *Arhgdia* | Q99PT1 | -1.08 | 0.028 |
| Ras-related protein Rab-1A | *Rab1A* | P62821 | -1.06 | 0.020 |
| ARF GTPase-activating protein GIT1 | *Git1* | Q68FF6 | 1.06 | 0.041 |
| GTP-binding protein Rheb | *Rheb* | Q921J2 | 1.06 | 0.038 |
| Guanine nucleotide-binding protein G(I)/G(S)/G(T)  subunit beta-2 | *Gnb2* | P62880 | 1.15 | 0.036 |
| RAS guanyl-releasing protein 2 | *Rasgrp2* | Q9QUG9 | 1.15 | 0.002 |
| Developmentally-regulated GTP-binding protein 1 | *Drg1* | P32233 | 1.16 | 0.032 |
|  |  |  |  |  |
| *Cytoskeletal and Cell Adhesion* |  |  |  |  |
| Myristoylated alanine-rich C-kinase substrate | *Marcks* | P26645 | -1.22 | 0.007 |
| Leukocyte surface antigen CD47 | *Cd47* | Q61735 | -1.17 | 0.011 |
| Basal cell adhesion molecule | *Bcam* | Q9R069 | -1.15 | 0.047 |
| Actin, alpha cardiac muscle 1 | *Actc1* | P68033 | -1.14 | 0.039 |
| Vimentin | *Vim* | P20152 | -1.14 | 0.032 |
| F-actin-capping protein subunit alpha-1 | *Capza1* | P47753 | -1.12 | 0.035 |
| Ensconsin | *Map7* | O88735 | -1.10 | 0.034 |
| Contactin-6 | *Cntn6* | Q9JMB8 | -1.10 | 0.032 |
| Prelamin-A/C | *Lmna* | P48678 | -1.09 | 0.023 |
| Neogenin | *Neo1* | P97798 | -1.08 | 0.022 |
| Plectin | *Plec* | Q9QXS1 | -1.07 | 0.025 |
| Microtubule-associated protein RP/EB family member 2 | *Mapre2* | Q8R001 | -1.07 | 0.034 |
| Protein Shroom2 | *Shroom2* | A2ALU4 | -1.06 | 0.036 |
| Contactin-1 | *Cntn1* | P12960 | -1.05 | 0.036 |
| Profilin-2 | *Pfn2* | Q9JJV2 | -1.04 | 0.022 |
| Neural cell adhesion molecule 1 | *Ncam1* | P13595 | -1.04 | 0.032 |
| Pericentriolar material 1 protein | *Pcm1* | Q9R0L6 | 1.06 | 0.049 |
| Neural Wiskott-Aldrich syndrome protein | *Wasl* | Q91YD9 | 1.07 | 0.002 |
| Protein phosphatase 1 regulatory subunit 12C | *Ppp1r12c* | Q3UMT1 | 1.09 | 0.004 |
| Neurochondrin | *Ncdn* | Q9Z0E0 | 1.12 | 0.049 |
| Microtubule-associated proteins 1A/1B light chain 3B | *Map1lc3b* | Q9CQV6 | 1.16 | 0.039 |
|  |  |  |  |  |
| *Transcription and Translation* |  |  |  |  |
| Small nuclear ribonucleoprotein F | *Snrpf* | P62307 | -1.31 | 0.013 |
| DBF4-type zinc finger-containing protein 2 homolog | *Zdbf2* | Q5SS00 | -1.19 | 0.015 |
| Histone H1.1 | *Hist1h1a* | P43275 | -1.15 | 0.029 |
| Polypyrimidine tract-binding protein 1 | *Ptbp1* | P17225 | -1.12 | 0.046 |
| D-tyrosyl-tRNA(Tyr) deacylase 1 | *Dtd1* | Q9DD18 | -1.12 | 0.035 |
| Histone H1.0 | *H1f0* | P10922 | -1.10 | 0.014 |
| 60S acidic ribosomal protein P1 | *Rplp1* | P47955 | -1.09 | 0.023 |
| 60S ribosomal protein L30 | *Rpl30* | P62889 | -1.09 | 0.009 |
| Transcription elongation factor A protein-like 5 | *Tceal5* | Q8CCT4 | -1.08 | 0.031 |
| Transcription activator BRG1 | *Smarca4* | Q3TKT4 | -1.07 | 0.046 |
| Seryl-tRNA synthetase, cytoplasmic | *Sars* | P26638 | -1.06 | 0.019 |
| 40S ribosomal protein S6 | *Rps6* | P62754 | -1.06 | 0.013 |
| Cytoplasmic FMR1-interacting protein 2 | *Cyfip2* | Q5SQX6 | 1.05 | 0.031 |
| Eukaryotic translation initiation factor 5A-1 | *Eif5a* | P63242 | 1.06 | 0.035 |
| Poly(rC)-binding protein 2 | *Pcbp2* | Q61990 | 1.06 | 0.034 |
| 60S ribosomal protein L12 | *Rpl12* | P35979 | 1.08 | 0.019 |
| TAR DNA-binding protein 43 | *Tardbp* | Q921F2 | 1.08 | 0.037 |
| SAP domain-containing ribonucleoprotein | *Sarnp* | Q9D1J3 | 1.08 | 0.033 |
| ELAV-like protein 3 | *Elavl3* | Q60900 | 1.08 | 0.025 |
| Eukaryotic translation initiation factor 3 subunit F | *Eif3f* | Q9DCH4 | 1.09 | 0.031 |
| Heterogeneous nuclear ribonucleoprotein F | *Hnrnpf* | Q9Z2X1 | 1.11 | 0.004 |
| NHP2-like protein 1 | *Nhp2l1* | Q9D0T1 | 1.11 | 0.044 |
| Poly [ADP-ribose] polymerase 1 | *Parp1* | P11103 | 1.11 | 0.020 |
| Transcription elongation regulator 1 | *Tcerg1* | Q8CGF7 | 1.13 | 0.008 |
| Heterogeneous nuclear ribonucleoprotein H2 | *Hnrnph2* | P70333 | 1.14 | 0.006 |
| General transcription factor II-I | *Gtf2i* | Q9ESZ8 | 1.15 | 0.049 |
| Heterogeneous nuclear ribonucleoprotein L-like | *Hnrpll* | Q921F4 | 1.17 | 0.021 |
| 60S ribosomal protein L18a | *Rpl18a* | P62717 | 1.23 | 0.043 |
| Phosphoribosylformylglycinamidine synthase | *Pfas* | Q5SUR0 | 1.34 | 0.014 |
|  |  |  |  |  |
| *Vesicles/Protein Transport* |  |  |  |  |
| Vacuolar protein-sorting-associated protein 36 | *Vps36* | Q91XD6 | -1.13 | 0.022 |
| Charged multivesicular body protein 6 | *Chmp6* | P0C0A3 | -1.13 | 0.025 |
| Transportin-1 | *Tnpo1* | Q8BFY9 | -1.11 | 0.013 |
| Transportin-2 | *Tnpo2* | Q99LG2 | -1.11 | 0.028 |
| Syntaxin-1B | *Stx1b* | P61264 | -1.11 | 0.017 |
| Golgi resident protein GCP60 | *Acbd3* | Q8BMP6 | -1.10 | 0.021 |
| Large neutral amino acids transporter small subunit 1 | *Slc7a5* | Q9Z127 | -1.09 | 0.049 |
| Gamma-soluble NSF attachment protein | *Napg* | Q9CWZ7 | -1.09 | 0.029 |
| Myosin-9 | *Myh9* | Q8VDD5 | -1.09 | 0.031 |
| Syntaxin-1A | *Stx1a* | O35526 | -1.08 | 0.036 |
| Vesicle-trafficking protein SEC22b | *Sec22b* | O08547 | -1.08 | 0.013 |
| Vacuolar-sorting protein SNF8 | *Snf8* | Q9CZ28 | -1.07 | 0.010 |
| Calnexin | *Canx* | P35564 | -1.07 | 0.032 |
| Myosin-10 | *Myh10* | Q61879 | -1.07 | 0.008 |
| Vacuolar protein sorting-associated protein 18 homolog | *Vps18* | Q8R307 | -1.06 | 0.031 |
| Transitional endoplasmic reticulum ATPase | *Vcp* | Q01853 | -1.03 | 0.033 |
| Mitochondrial carrier homolog 2 | *Mtch2* | Q791V5 | 1.08 | 0.009 |
| Sorting nexin-16 | *Snx16* | Q8C080 | 1.09 | 0.026 |
| Vacuolar protein sorting-associated protein 29 | *Vps29* | Q9QZ88 | 1.14 | 0.002 |
| Tyrosine-protein phosphatase non-receptor type 23 | *Ptpn23* | Q6PB44 | 1.15 | 0.010 |
| Vacuolar protein sorting-associated protein VTA1 homolog | *Vta1* | Q9CR26 | 1.18 | 0.017 |
|  |  |  |  |  |
| *Others* |  |  |  |  |
| Quinone oxidoreductase-like protein 1 | *Cryzl1* | Q921W4 | -1.18 | 0.047 |
| PRA1 family protein 3 | *Arl6ip5* | Q8R5J9 | -1.09 | 0.038 |
| Thioredoxin-like protein 1 | *Txnl1* | Q8CDN6 | -1.09 | 0.024 |
| 4-trimethylaminobutyraldehyde dehydrogenase | *Aldh9a1* | Q9JLJ2 | -1.08 | 0.034 |
| Collagen type IV alpha-3-binding protein | *Col4a3bp* | Q9EQG9 | -1.07 | 0.008 |
| Secernin-1 | *Scrn1* | Q9CZC8 | -1.07 | 0.023 |
| Sodium/calcium exchanger 1 | *Slc8a1* | P70414 | -1.07 | 0.044 |
| Long-chain fatty acid transport protein 4 | *Slc27a4* | Q91VE0 | 1.05 | 0.045 |
| Transmembrane and TPR repeat-containing protein 3 | *Tmtc3* | Q8BRH0 | 1.08 | 0.029 |
| Titin | *Ttn* | A2ASS6 | 1.09 | 0.016 |
| Integral membrane protein 2C | *Itm2c* | Q91VK4 | 1.09 | 0.045 |
| Redox-regulatory protein PAMM | *Fam213a* | Q9CYH2 | 1.09 | 0.030 |
| Uncharacterized membrane protein C1orf95 homolog | *Stum* | Q0VBF8 | 1.10 | 0.028 |
| Constitutive coactivator of PPAR-gamma-like protein 2 | *Fam120c* | Q8C3F2 | 1.10 | 0.048 |
| NmrA-like family domain-containing protein 1 | *Nmral1* | Q8K2T1 | 1.13 | 0.032 |

^a^Fold changes between males and females. Positive values represent male-biased proteins and negative values represent female-biased proteins.

^b^Proteins differentially expressed (p ≤ 0.05) in males and females.
