# Supplementary material for: Proteomic analyses of limbic regions in neonatal male, female and androgen receptor knockout mice
Source: BMC Neurosci. 2017 Jan 5;18:9. doi: 10.1186/s12868-016-0332-1 (PMC5217640; doi:10.1186/s12868-016-0332-1)
Supplement: Supplementary file 2 — Additional file 2: Table S2. Differentially expressed proteins in amygdala and hypothalamus of 8-day-old male and ARNesDel male mice. [file 12868_2016_332_MOESM2_ESM.docx]

**Table S2.** Differentially expressed proteins in amygdala and hypothalamus

of 8-day-old male and AR^NesDel^ male mice.

| **Proteins** | **Gene**  **symbol** | **Uniprot Accession Nr** | **Fold change^a^** | **p-value^b^** |
| --- | --- | --- | --- | --- |
| *Scaffolders and Adaptors* |  |  |  |  |
| 14-3-3 protein eta | *Ywhah* | P68510 | -1.08 | 0.048 |
| 14-3-3 protein theta | *Ywhaq* | P68254 | -1.03 | 0.034 |
|  |  |  |  |  |
| *Signalling Molecules and Enzymes* |  |  |  |  |
| Protein sel-1 homolog 1 | *Sel1l* | Q9Z2G6 | -1.11 | 0.028 |
| Hypoxia up-regulated protein 1 | *Hyou1* | Q9JKR6 | -1.09 | 0.016 |
| Dystrobrevin alpha | *Dtna* | Q9D2N4 | -1.09 | 0.041 |
| N(G),N(G)-dimethylarginine dimethylaminohydrolase 1 | *Ddah1* | Q9CWS0 | -1.08 | 0.003 |
| L-lactate dehydrogenase B chain | *Ldhb* | P16125 | -1.05 | 0.041 |
| Heat shock 70 kDa protein 4 | *Hspa4* | Q61316 | -1.04 | 0.009 |
| Fatty acid synthase | *Fasn* | P19096 | 1.04 | 0.048 |
| NADH dehydrogenase [ubiquinone] iron-sulfur protein 5 | *Ndufs5* | Q99LY9 | 1.05 | 0.023 |
| CCA tRNA nucleotidyltransferase 1, mitochondrial | *Trnt1* | Q8K1J6 | 1.09 | 0.037 |
| Breakpoint cluster region protein | *Bcr* | Q6PAJ1 | 1.12 | 0.035 |
| Protein CutA | *Cuta* | Q9CQ89 | 1.16 | 0.040 |
|  |  |  |  |  |
| *Ser/Thr Kinases* |  |  |  |  |
| Calcium/calmodulin-dependent protein kinase  type II subunit gamma | *Camk2g* | Q923T9 | -1.08 | 0.002 |
| BR serine/threonine-protein kinase 1 | *Brsk1* | Q5RJI5 | 1.09 | 0.025 |
|  |  |  |  |  |
| *G-proteins and Modulators* |  |  |  |  |
| Golgin subfamily A member 2 | *Golga2* | Q921M4 | 1.07 | 0.045 |
| Rho GTPase-activating protein 39 | *Arhgap39* | P59281 | 1.12 | 0.000 |
|  |  |  |  |  |
| *Cytoskeletal and Cell Adhesion* |  |  |  |  |
| Actin, alpha cardiac muscle 1 | *Actc1* | P68033 | -1.20 | 0.027 |
|  |  |  |  |  |
| *Transcription and Translation* |  |  |  |  |
| Intracellular hyaluronan-binding protein 4 | *Habp4* | Q9JKS5 | -1.15 | 0.026 |
| Tryptophanyl-tRNA synthetase, cytoplasmic | *Wars* | P32921 | -1.07 | 0.015 |
| Splicing factor 3A subunit 2 | *Sf3a2* | Q62203 | -1.05 | 0.048 |
| 40S ribosomal protein SA | *Rpsa* | P14206 | 1.05 | 0.041 |
| Bifunctional aminoacyl-tRNA synthetase | *Eprs* | Q8CGC7 | 1.06 | 0.038 |
| Transcription elongation factor B polypeptide 1 | *Tceb1* | P83940 | 1.07 | 0.015 |
| KH domain-containing, RNA-binding,  signal transduction-associated protein 3 | *Khdrbs3* | Q9R226 | 1.10 | 0.035 |
| CUGBP Elav-like family member 2 | *Celf2* | Q9Z0H4 | 1.16 | 0.036 |
|  |  |  |  |  |
| *Vesicles/Protein Transport* |  |  |  |  |
| Dynein light chain 1, cytoplasmic | *Dynll1* | P63168 | -1.17 | 0.034 |
| Vesicle-associated membrane protein-  associated protein B | *Vapb* | Q9QY76 | -1.11 | 0.023 |
| Protein transport protein Sec61 subunit beta | *Sec61b* | Q9CQS8 | -1.11 | 0.040 |
| Charged multivesicular body protein 6 | *Chmp6* | P0C0A3 | -1.10 | 0.028 |
| B-cell receptor-associated protein 29 | *Bcap29* | Q61334 | -1.09 | 0.027 |
| Vesicle-associated membrane protein 2 | *Vamp2* | P63044 | -1.06 | 0.014 |
|  |  |  |  |  |
| *Others* |  |  |  |  |
| 6.8 kDa mitochondrial proteolipid | *Mp68* | P56379 | -1.12 | 0.009 |
| Phytanoyl-CoA hydroxylase-interacting protein-like | *Phyhipl* | Q8BGT8 | -1.11 | 0.035 |
| Malectin | *Mlec* | Q6ZQI3 | -1.08 | 0.042 |
| UPF0480 protein C15orf24 homolog | *ORF3* | Q9EP72 | 1.06 | 0.042 |
| Fatty acid-binding protein, heart | *Fabp3* | P11404 | 1.09 | 0.037 |
| Protein unc-119 homolog A | *Unc119* | Q9Z2R6 | 1.23 | 0.027 |

^a^Fold changes between males and AR^NesDel^ males. Positive values represent male-biased proteins and negative values represent AR^NesDel^ male-biased proteins.

^b^Proteins differentially expressed (p ≤ 0.05) in males and AR^NesDel^ males.
